# Analysis of body condition indices reveals different ecotypes of the Antillean manatee

**DOI:** 10.1038/s41598-021-98890-0

**Published:** 2021-09-30

**Authors:** D. N. Castelblanco-Martínez, D. H. Slone, S. S. Landeo-Yauri, E. A. Ramos, A. Alvarez-Alemán, F. L. N. Attademo, C. A. Beck, R. K. Bonde, S. M. Butler, L. J. Cabrias-Contreras, D. Caicedo-Herrera, J. Galves, I. V. Gómez-Camelo, D. González-Socoloske, D. Jiménez-Domínguez, F. O. Luna, Y. Mona-Sanabria, J. B. Morales-Vela, L. D. Olivera-Gómez, J. A. Padilla-Saldívar, J. Powell, J. P. Reid, G. Rieucau, A. A. Mignucci-Giannoni

**Affiliations:** 1 Consejo Nacional de Ciencia y Tecnología, Mexico city, Mexico; 2 Universidad de Quintana Roo, Chetumal, Mexico; 3 Fundación Internacional Para la Naturaleza y la Sustentabilidad, Chetumal, Mexico; 4 U.S. Geological Survey, Sirenia Project, Wetland and Aquatic Research Center, Gainesville, USA; 5 Clearwater Marine Aquarium Research Institute, Clearwater, USA; 6 Centro de Investigaciones Marinas, Universidad de la Habana, Havana, Cuba; 7 Instituto Chico Mendes de Conservação da Biodiversidade/Centro Nacional de Pesquisa e Conservação de Mamíferos Aquáticos, Pernambuco, Brazil; 8 Caribbean Manatee Conservation Center, Inter American University of Puerto Rico, Bayamon, Puerto Rico; 9 Fundación Omacha, Bogotá, Colombia; 10 Andrews University, Berrien Springs, USA; 11 Universidad Juárez Autónoma de Tabasco, Villahermosa, Mexico; 12 El Colegio de La Frontera Sur, Chetumal, Mexico; 13 Louisiana Universities Marine Consortium, Chauvin, USA; 14 Center for Conservation Medicine and Ecosystem Health, Ross University School of Veterinary Medicine, Bassetterre, Saint Kitts and Nevis

**Keywords:** Ecology, Behavioural ecology, Conservation biology, Evolutionary ecology

## Abstract

Assessing the body condition of wild animals is necessary to monitor the health of the population and is critical to defining a framework for conservation actions. Body condition indices (BCIs) are a non-invasive and relatively simple means to assess the health of individual animals, useful for addressing a wide variety of ecological, behavioral, and management questions. The Antillean manatee (*Trichechus manatus manatus*) is an endangered subspecies of the West Indian manatee, facing a wide variety of threats from mostly human-related origins. Our objective was to define specific BCIs for the subspecies that, coupled with additional health, genetic and demographic information, can be valuable to guide management decisions. Biometric measurements of 380 wild Antillean manatees captured in seven different locations within their range of distribution were obtained. From this information, we developed three BCIs (BCI_1_ = UG/SL, BCI_2_ = W/SL^3^, BCI_3_ = W/(SL*UG^2^)). Linear models and two-way ANCOVA tests showed significant differences of the BCIs among sexes and locations. Although our three BCIs are suitable for Antillean manatees, BCI_1_ is more practical as it does not require information about weight, which can be a metric logistically difficult to collect under particular circumstances. BCI_1_ was significantly different among environments, revealing that the phenotypic plasticity of the subspecies have originated at least two ecotypes—coastal marine and riverine—of Antillean manatees.

## Introduction

The nutritional status of wild animals is an important factor defining individual survival, influencing growing rates, reproductive frequency and fecundity^1^, and has population-level consequences^2^. Seasonal and spatial variation in body condition—a key variable to infer energetic reserves—provides insight into animal foraging success over time and space, and enables inferences regarding aspects of the ecosystem’s health and of the population’s resilience^3^. Developing reliable, non-invasive means to assess body condition is important to clarify many life-history and ecological characteristics of wild populations, as well as to guide decisions for successful management in captivity.

Condition indices are defined as biochemical, physiological, or morphological metrics used to define the health of individuals and are assumed to be related to foraging success and ultimately fitness^4^. Thus, they can be a simple and sensible tool to detect differences among ill and healthy individuals^5^. Some of these condition indices are based on the measurement of biological macromolecules including lipids, nucleic acids, and proteins^6^; or physiological parameters such as plasma-lipid metabolites, hormone levels, and hematological levels^7^. Another group of indices are biometric in nature^8^ and are used as a proxy for energy reserves, nutrient reserves, or mass of body fat^9,10^. According to the method to obtain them, BCIs generally fall into two categories: ratio indices (*i.e.*, ratio of body mass divided by body length) and residual indices (*i.e*., residuals from regression of body mass on body length)^9^.

The evaluation of BCIs have been applied to a broad array of questions in monitoring long-term effects including: to assess the fluctuation of availability of feeding resources^11^; to explore the impact of parasitic and infectious diseases on individual health^12,13^; to evaluate the consequences of metal bioaccumulation^14^, organic pollutants^15^, and habitat fragmentation^16^; and to infer reproductive state in females^10^ and males^17^. BCIs have been developed for several marine mammals including polar bears *Ursus maritimus*^18^, Pacific walruses *Odobenus rosmarus divergens*^19^, fur seals *Callorhinus ursinus*^20^, Steller sea lions *Eumetopias jubatus*^21^, cetaceans^3^, dugongs *Dugong dugon*^17^, and Florida manatees *Trichechus manatus latirostris*^22^.

Manatees (Trichechidae) are herbivorous, fully aquatic mammals of the order Sirenia and include three extant species: the Amazonian manatee *Trichechus inunguis*, the African manatee *T. senegalensis*, and the West Indian manatee *T. manatus*. The two recognized subspecies of the West Indian manatee are the Florida manatee *T. m. latirostris* and the Antillean manatee *T. m. manatus*, the latter distributed in the Gulf of Mexico, the Caribbean, and Atlantic Ocean from northern Mexico (Tamaulipas State) to the northeastern coast of Brazil (Alagoas State), including the Greater Antilles. Across its range of distribution, the subspecies faces a number of human-related threats including poaching, entanglement in fishing nets, boat collisions, and habitat fragmentation or loss^23^. Because of this, coupled with slow reproductive and population growth rates, the Antillean manatee is considered endangered by the IUCN, and protected by local laws in almost every country of its distribution^24^. The subspecies inhabits many diverse marine, estuarine, and riverine habitats^25^, moving and behaving differently depending on the environment. Despite the wide distribution and plasticity of Antillean manatees to adapt to different habitats, little genetic, morphological, or physiological evidence currently supports the distinctiveness of ‘ecotypes’ (*e.g*. several expressions of the same population resulting from local adaptation to heterogeneous environmental conditions^26^).

Harshaw et al*.* (2016) determined a normal range of biometric BCIs for Florida manatees, and explored differences in manatee body condition between geographic areas. The authors suggested that these indices can be useful for monitoring manatees in captivity, and to also serve as baseline for the wild Florida manatee population. However, those indices are likely unsuitable in studies of Antillean manatees because *T. m. latirostris* has a larger, stockier body shape than *T. m. manatus*^27^, and the growth rate between the subspecies differs^28^. Here, we analyze three morphometric body condition indices for Antillean manatees from several areas of the subspecies’ distribution, after the indices found in Harshaw et al*.* (2016). We compare the results of the three BCIs across geographic location, sex, and habitat type to identify differences and similarities.

## Methods

Biometric data of wild Antillean manatees were obtained from individuals captured during long-term projects conducted in different regions of the subspecies’ distribution, or during procedures of rescue and relocation. The field procedures were performed in accordance with international and national guidelines and regulations, following rigorous ethical standards to ensure the welfare of study manatees and the protection of their habitats. All the proposed protocols for manatee capture, restriction, measurement, and sample collection were evaluated and approved by special licensing committees of each of the following entities: CITMA (Ministerio de Ciencia Tecnologia y Medio Ambiente, Cuba), USFWS (U.S. Fish and Wildlife Service, Puerto Rico and Guantanamo in Cuba), SEMARNAT (Secretaría de Medio Ambiente y Recursos Naturales, Mexico), BDF (Belize Department of Forestry, Belize), MMADS (Ministerio de Medio Ambiente y Desarrollo Sostenible, Colombia), and SISBIO (Sistema de Autorização e Informação em Biodiversidade, Brazil). The captures were conducted under research permits issued by the local environmental authority of each country (See Acknowledgment section for permit details).

**Figure 1:**
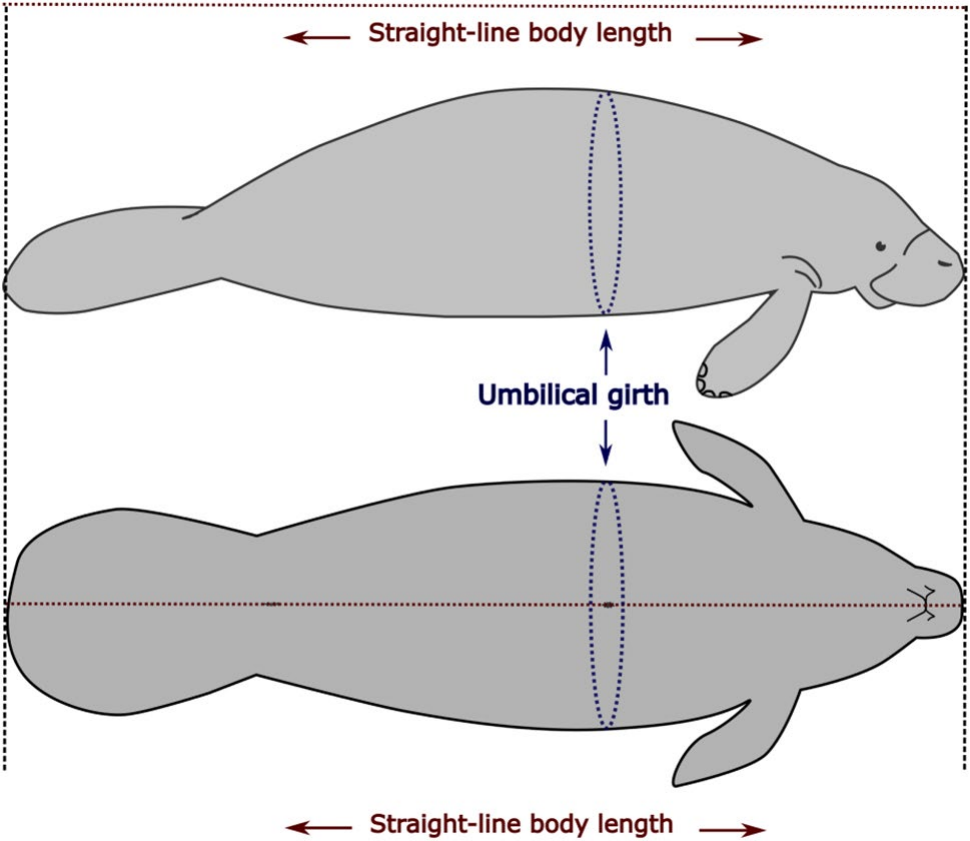
Illustration of straight-line total length (SL) and umbilical girth (UG) measured in wild and captive Antillean manatees.

These projects focused on the condition of manatees and involved health assessments and very often satellite telemetry monitoring. Information about each manatee’s individual identification, date, location, sex, and collector were also included in the database. Maps of the study locations and that of each captured wild manatee were created in QGIS 3.14^29^. Straight-line body length (SL) was measured from the tip of the snout in a relaxed position to the median notch of the tail, and the body circumference was measured at the level of the umbilical scar (umbilical girth, UG) (Fig. 1). Where possible each manatee was weighed using a stretcher suspended from a crane scale^30^. Manatee age classes were classified as calves (< 175 cm), subadults (175–225 cm), or adults (> 225 cm)^31^.

We standardized a qualitative scale of body appearance for each manatee (field score) in order to discard from the analysis any individual with evident abnormal health status, and all suspected pregnant females. The standardization was based on the visual assessment of manatee bodies at the moment of biometric data collection, the animal’s condition was categorized as emaciated (C1), thin (C2), ideal (C3), overweight (C4), or obese (C5) (Supplementary material 1 and 2). Some manatees were graded using a simplified scale that combined C1-2 and C4-5 field scores, so data from the first two groups (C1, C2) and data from the last two groups (C4, C5) were combined to analyze whether field score was significantly correlated with BCI. These groupings were not used for the final BCI analysis. Animals that were not visually scored were not included in the field score analysis, but were included in the final BCI analysis. Since body morphometrics are influenced by gestational stage^17^, females at the last stage of pregnancy were also excluded. Of the large number of measurements taken during manatee health assessments, we only used data on body mass (W), umbilical girth (UG), and straight-line total length (SL) for these analyses. The first BCI (Eq. 1) represents the expected proportionality among umbilical girth (UG) and straight-line total length (SL), assuming a geometric similarity (b = 1)^22^:

1 $${\mathrm{BCI}}_{1}=\frac{\mathrm{UG}}{{\mathrm{SL}}^{\mathrm{b}1}}$$

Geometric similarity among all animal sizes, that is larger animals having the same relative shape as smaller animals, would result in *b*_*1*_ = 1. If longer animals have a proportionately larger girth, *b*_*1*_ > 1, and *b*_*1*_ < 1 for the opposite relationship. Higher BCI_1_ values indicate a population with proportionally larger girths.

The allometric relationship between the weight (W) and the SL of a manatee is the second BCI (Eq. 2), expressed as:

2 $${BCI}_{2}=\frac{W}{{SL}^{b2}}$$

If the weight of manatees is proportional among all sizes, *b*_*2*_ = 3. If longer animals have a proportionately higher weight, *b*_*2*_ > 3, and *b*_*2*_ < 3 for the opposite relationship. Here, higher BCI_2_ values indicate a population of proportionally heavier weight at a given SL.

The final BCI represents the allometric relationship among all three measurements, measuring the ratio of W to the two measured dimensions SL and UG (Eq. 3 ${\mathrm{BCI}}_{1}=\frac{\mathrm{UG}}{{\mathrm{SL}}^{\mathrm{b}1}}$):

3 $${BCI}_{3}=\frac{W}{{SL\times UG}^{b3}}$$

Here, geometrically similar animals would result in *b*_*3*_ = 2. If animals with a given SL and UG have a proportionately higher weight, *b*_*3*_ > 2, and *b*_*3*_ < 2 for the opposite relationship. Therefore, higher BCI scores indicate a population of heavier weight individuals at a given SL and UG. With all three measurements being incorporated, BCI_3_ has the potential to be more accurate than the other types, but it also may mask morphological differences among populations because animals that are long and thin may have a similar BCI_3_ as animals that are shorter and stouter.

Initial data exploration suggested that outlier data points were present, so all analyses were performed with robust models in R^32,33^. First, each BCI formula was fit to the available data to determine the overall BCI and *b* coefficient using the nonlinear model function *nlrob* (package *robustbase*^34^)*.* To test for geometric similarity among different sizes of manatees, the following reformulations of Eqs. (1)–(3) were fit: for BCI_1_:

4 $$UG= {BCI}_{1}{\times SL}^{(1-{b}_{1})}$$

for BCI_2_:

5 $$W= {BCI}_{2}{\times SL}^{(3-{b}_{2})}$$

and for BCI_3_:

6 $$W= {BCI}_{3}{\times SL\times UG}^{(2-{b}_{3})}.$$

Two of the factors (Habitat and Sex) were also included in a non-linear robust model for each of the BCI equations to determine their effect on BCI and *b*. For example, the BCI_1_ equation was:

7 $$UG= {(BCI}_{1}+{r}_{1}\times Riverine+{m}_{1}\times Male){\times SL}^{({b}_{1}+{br}_{1}\times Riverine+{bm}_{1}\times Male)}.$$

The *b* parameters that were fit in Eqs. (4)–(6) were incorporated into Eqs. (1)–(3) to calculate each BCI for each manatee. This removed the non-linear component and allowed us to fit each BCI in linear robust models using function *lmrob* (package *robustbase*) with settings = "KS2014"^35^ to estimate the effect of the factor variables, including Country (Mexico was split into Chetumal Bay and Gulf Coast locations), habitat type (Marine or Riverine), sex (F, M), and field condition rating (C1-2, C3, or C4-5). For the latter test the thin and obese animal data were re-incorporated into the data set. These models were of the form:

8 $$BCI=a+c\times Country+h\times Habitat+s\times Sex+f\times FieldCondition$$

Data visualization was performed with the package *ggplot2*^36^.

## Results

**Figure 2:**
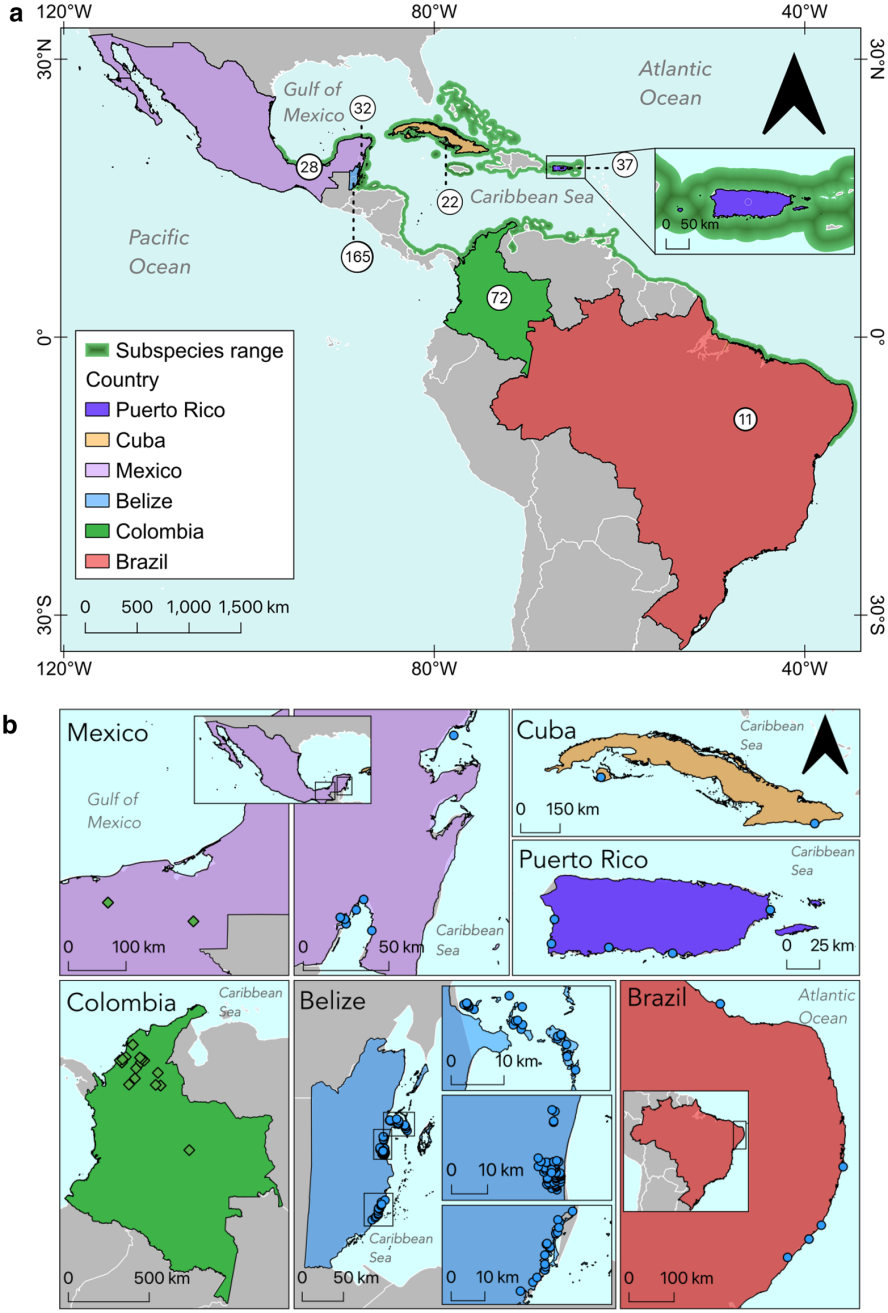
(**a**). Map of the countries where samples of Antillean manatees were collected overlaid with a map of their distribution^24^ with number of individuals shown in circles. (**b**). Specific locations of manatee captures. Manatees were captured in coastal marine (blue circles) and riverine (green diamonds) environments.

Records were obtained from 416 wild Antillean manatees, of which 380 had data for body mass (W), umbilical girth (UG), and/or straight-line total length (SL). Of these, 362 individuals had good (C3) or unscored body condition (182 females, 184 males), which were assumed to have a healthy appearance. The manatees were captured or rescued between 1978 to 2019 in Puerto Rico (*n* = 37), Cuba (*n* = 22), Mexico (southern Gulf of Mexico, Mexico G:* n* = 28; Mexican Caribbean, Mexico C:* n* = 32), Belize (*n* = 160), Colombia (*n* = 72), and Brazil (*n* = 11) (Table 1, Fig. 2). The remaining individuals were scored emaciated or thin, hereafter termed “thin” (C1 or C2; *n* = 5); or overweight or obese, hereafter termed “obese” (C4 or C5; *n* = 13) and were not used in the analyses except for those that specifically included a field-scored body condition. Not all measurements were made on every manatee, and as a result, sample sizes differed for each body condition index. There were 353 manatees with UG and SL measurements (BCI_1_), 234 with W and SL (BCI_2_), and 225 with all three measurements (BCI_3_).

**Table 1:** Details on sex and age class of wild Antillean manatees captured for health assessments or rescues in seven locations throughout their range. Mexico C = Mexican Caribbean, Mexico G = Southern Gulf of Mexico.

Country	Females	Males	Adults	Subadults	Calves	Total
Puerto Rico	19	18	25	8	4	37
Cuba	11	11	14	2	6	22
Mexico G	16	12	10	12	6	28
Mexico C	12	20	16	10	6	32
Belize	79	81	92	48	20	160
Colombia	36	36	14	34	24	72
Brazil	4	7	5	6	–	11
Total	**177**	**185**	**176**	**120**	**66**	**362**

Initial estimates of BCI by country showed a strong grouping by the predominant habitat type used by manatees in each location. Countries where manatees were captured predominately in coastal, marine, bay/lagoon, and estuarine habitats (Puerto Rico, Cuba, Mexico C, Belize, Brazil) had very similar values, and countries where manatees were captured in riverine habitats (Colombia, Mexico G) were also similar (Table 2). Including country and habitat types as factors in the *nlrob* model showed that country and type of coastal habitat were not significant factors, but coastal vs. riverine habitat was very significant. Therefore we incorporated this into the final models.

**Table 2:** BCI values fitted to wild Antillean manatees with non-linear robust regression by country of capture.

Habitat	Country	BCI_1_		BCI_2_		BCI_3_	
		Mean	SE	Mean	SE	Mean	SE
Riverine	Colombia	0.686	0.006	19.550	0.394	36.003	0.518
	Mexico G	0.695	0.009	22.032	0.557	38.186	0.717
Coastal marine	Belize	0.755	0.004	25.166	0.302	38.438	0.366
	Brazil	0.760	0.014	26.373	1.265	39.743	1.431
	Cuba	0.750	0.010	–	–	–	–
	Mexico C	0.758	0.008	23.571	0.526	35.901	0.633
	Puerto Rico	0.763	0.008	24.713	0.807	37.991	1.091

All *b* values were smaller than the corresponding geometric similarity values, indicating that longer manatees were proportionally thinner than shorter manatees (Table 3). This was especially apparent in *b*_*1*_, which was 7% smaller in females and 15% smaller in males, and *b*_*2*_, which was 12% smaller in females and 15% smaller in males. The *b*_*3*_ estimate was 6% smaller in both males and females, but was not significant. For this reason, the *b* parameter was fitted in each model rather than use the theoretical values for geometric similarity. The *b* values for the Riverine manatees were not significantly different from those of the Coastal manatees.

**Table 3:** BCI and *b* values fitted to wild Antillean manatees with non-linear robust regression. (* = p < 0.05; ** = p < 0.005; *** = p < 0.0005).

Parameter	Mean	SE	t value	Pr( >|t|)
BCI_1_ (Female, Coastal)	0.765	0.025	–	–
BCI_1_ (Male, Coastal)	0.801	0.026	–	–
BCI_1_ (Female–Male)	– 0.036	0.030	– 1.201	0.2304
BCI_1_ (Coastal–Riverine)	0.078	0.034	2.288	0.0227*
1-b_1_ (Female, Coastal)	0.074	0.035	2.107	0.0358*
1-b_1_ (Male, Coastal)	0.154	0.035	4.378	< 0.0001***
b_1_ (Female–Male)	0.080	0.042	1.887	0.0599
b_1_ (Coastal–Riverine)	– 0.007	0.053	– 0.141	0.8881
BCI_2_ (Female, Coastal)	25.043	2.303	–	–
BCI_2_ (Male, Coastal)	25.963	2.309	–	–
BCI_2_ (Female–Male)	– 0.920	1.676	– 0.549	0.5837
BCI_2_ (Coastal–Riverine)	8.385	2.874	2.918	0.0039**
3-b_2_ (Female, Coastal)	0.374	0.093	4.016	< 0.0001***
3-b_2_ (Male, Coastal)	0.439	0.092	4.784	< 0.0001***
b_2_ (Female–Male)	0.065	0.074	0.877	0.3814
b_2_ (Coastal–Riverine)	– 0.277	0.143	– 1.944	0.0532
BCI_3_ (Female, Coastal)	37.600	2.125	–	–
BCI_3_ (Male, Coastal)	37.965	2.292	–	–
BCI_3_ (Female–Male)	– 0.365	2.539	– 0.144	0.8859
BCI_3_ (Coastal–Riverine)	1.668	2.738	0.609	0.5431
2-b_3_ (Female, Coastal)	0.112	0.087	1.277	0.2029
2-b_3_ (Male, Coastal)	0.117	0.098	1.190	0.2353
b_3_ (Female–Male)	0.005	0.110	0.050	0.9603
b_3_ (Coastal–Riverine)	– 0.113	0.131	– 0.860	0.3905

**Figure 3:**
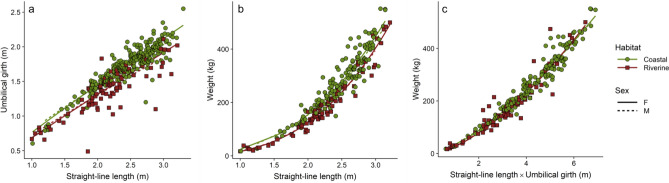
Relationships between: (**a**) Umbilical Girth (UG) and Straight-line length (SL) (*n* = 353); (**b**) Weight (W) and Straight-line length (SL) (*n* = 234); and (**c**) Weight (W) and Straight-line length (SL) X Umbilical girth (UG) (*n* = 225), from two habitat types. Lines indicate fit from a nonlinear robust regression.

**Figure 4:**
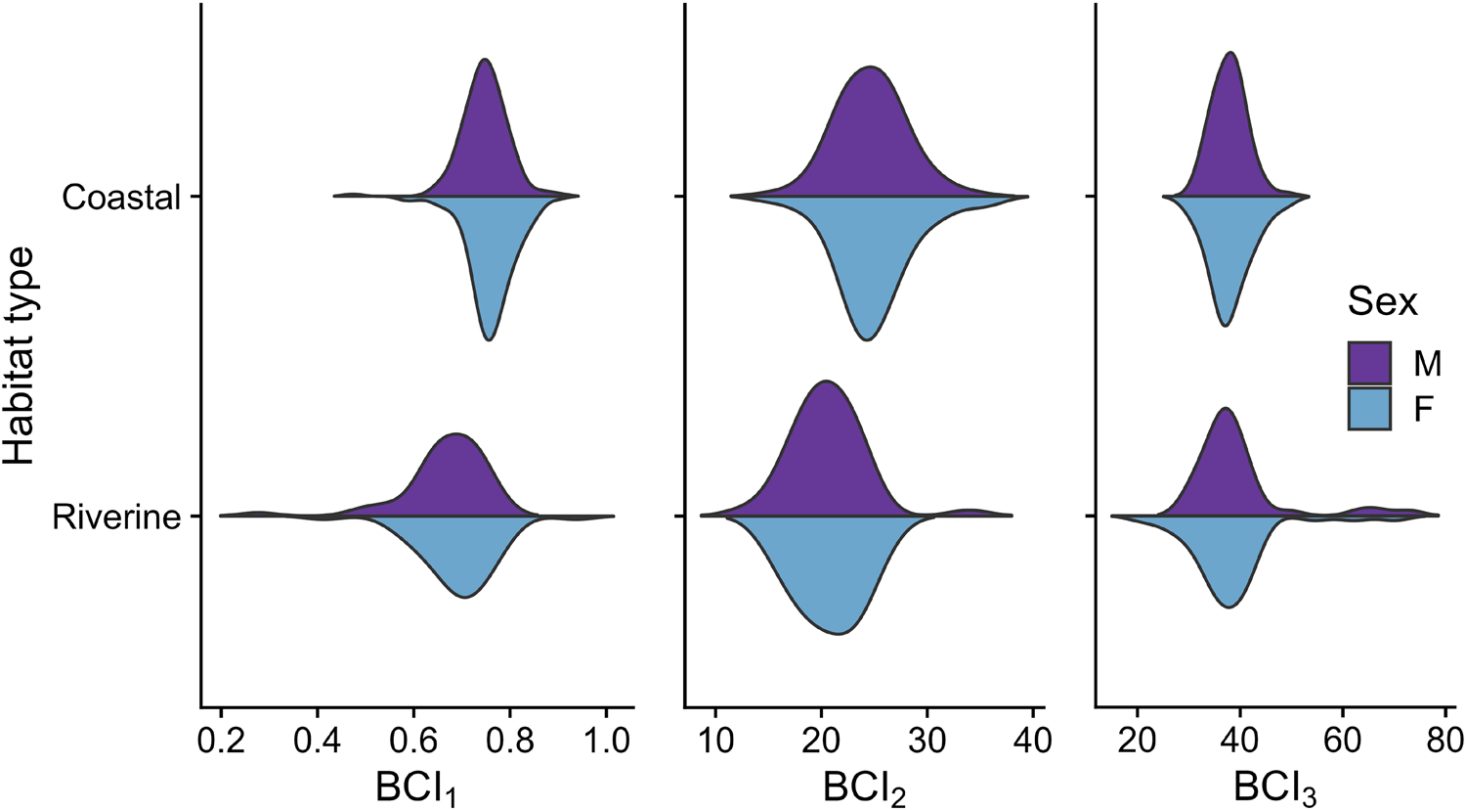
Violin plots showing the distribution of the three body condition indices: BCI_1_ (UG/SL^b1^), BCI_2_ (W/SL^b2^), and BCI_3_ (W/[SL × UG^b3^]) obtained from wild Antillean manatees (*n* = 362) in two habitat types. The horizontal axis of each violin represents the value of the obtained BCI. The shape of the violin plot depicts the distribution of the values of the BCI in each location and for each sex. UG = umbilical girth, W = weight, SL = straight body length.

**Figure 5:**
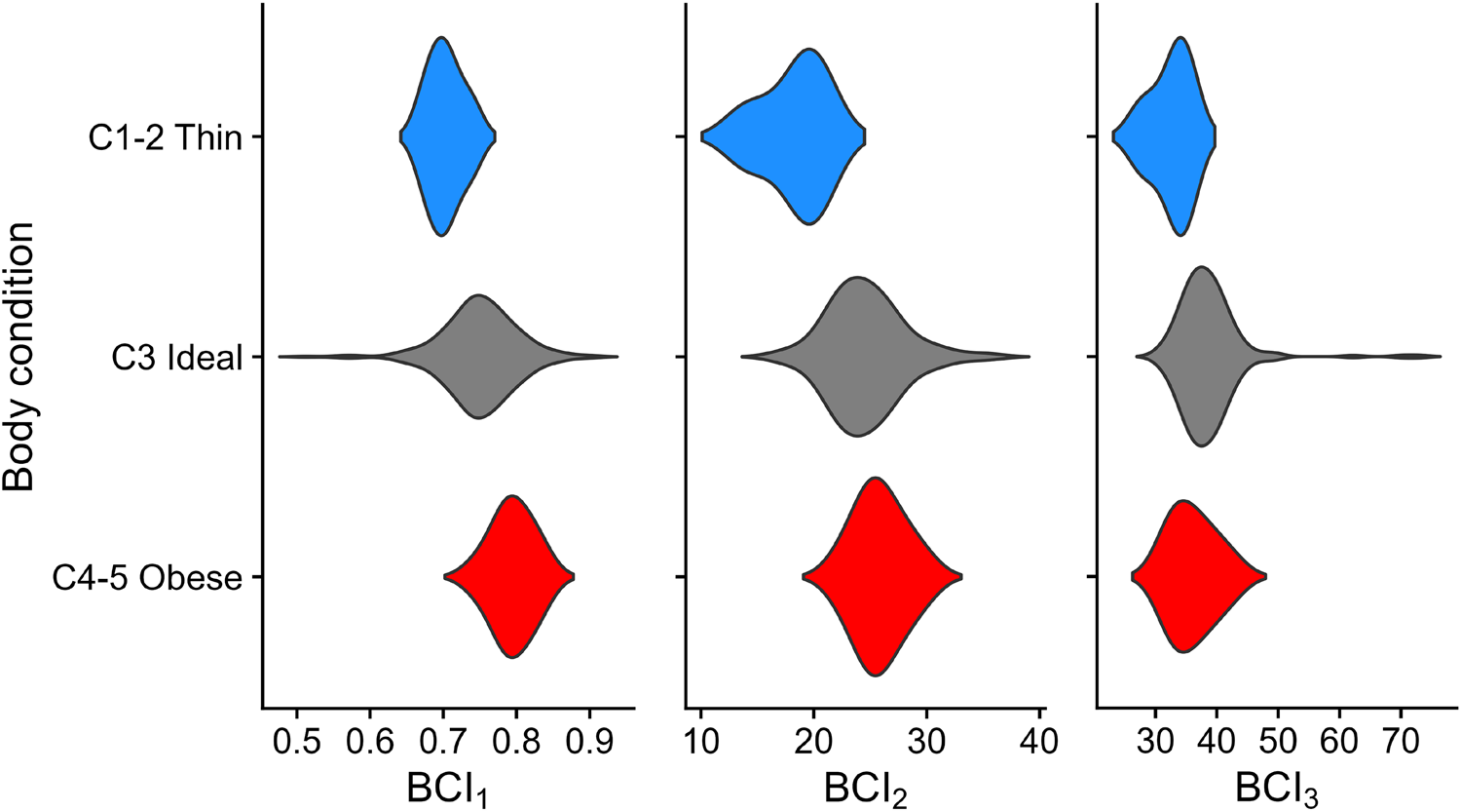
Violin plots showing the distribution of the three body condition indices: BCI_1_ (UG/SL^b1^), BCI_2_ (W/SL^b2^), and BCI_3_ (W/[SL × UG^b3^]) obtained from wild Antillean manatees (*n* = 362) that were classified in the field as thin (C1 or C2; n = 5), ideal body condition (C3; n = 287), or obese (C4 or C5; n = 13). The horizontal axis of each violin represents the value of the obtained BCI. The shape of the violin plot depicts the distribution of the values of the BCI of each body type classification. UG = umbilical girth, W = weight, SL = straight body length.

The BCI values for Male vs. Female manatees did not differ significantly, but the BCI_1_ and BCI_2_ of Coastal manatees were both larger than those of the Riverine animals, indicating an overall larger girth and higher weight for a given body length (Table 3). Both BCI_1_ and BCI_2_ showed a strong effect from Habitat, and a weak, inconsistent effect from Sex. Animals from the Riverine habitat found in Colombia and Mexico G were consistently thinner and lighter than their Coastal counterparts, even those from nearby countries. Conversely, the BCI_3_ and *b*_3_ values were very consistent across Habitat and Sex. By incorporating girth and length, this measure was robust to environmental and genetic heterogeneity and provided the most accurate size to weight relationship (Figs. 3, 4). Including the thin (C1 and C2) and obese (C4 and C5) animals into the models showed that all BCI measurements were significantly different for the thin animals, but only BCI_1_ was significantly different in the obese animals (Table 4). It is notable that most of the manatees that were field-classified as emaciated/thin, and all of the manatees classified as obese were well within the minimum and maximum values for the “normal” weight manatees (i.e. C3), even after accounting for Sex and Habitat (Fig. 5).

**Table 4:** BCI and *b* values fitted to wild Antillean manatees with non-linear robust regression. (* = p < 0.05; ** = p < 0.005; *** = p < 0.0005).

Parameter	Mean	SE	t value	Pr( >|t|)
BCI_1_ (Thin C1,2–Ideal C3)	– 0.059	0.023	– 2.530	0.0118*
BCI_1_ (Obese C4,5–Ideal C3)	0.041	0.015	2.703	0.0072**
BCI_2_ (Thin C1,2–Ideal C3)	– 5.658	1.393	– 4.062	< 0.0001***
BCI_2_ (Obese C4,5–Ideal C3)	1.047	0.748	1.399	0.1632
BCI_3_ (Thin C1,2–Ideal C3)	– 4.425	1.460	– 3.031	0.0027**
BCI_3_ (Obese C4,5–Ideal C3)	– 1.167	0.795	– 1.468	0.1434

All the BCIs for males from both habitat types obtained in this study were on average smaller than those obtained from Florida manatees (Table 5)^22^. All of the Female BCIs from the Riverine habitat type were on average smaller than those from Florida, but the Antillean manatee BCI_1_ and BCI_2_ from the Coastal habitat were larger.

**Table 5:** Body condition indices (BCI) for West Indian manatees *Trichechus manatus.* W = body mass, UG = umbilical girth, SL = straight-line total length.

	Females					**N**	***B***	Males		
	N	*b*	BCI					BCI		
			Mean	SD	Range			Mean	SD	Range
**Antillean manatee** (***T. m. manatus***)*—Coastal marine										
BCI_1_ = UG/SL^b1^	127	0.926	0.76	0.05	0.58–0.90	136	0.846	0.75	0.05	0.47–0.90
BCI_2_ = W/SL^b2^	70	2.626	25.0	3.75	14.8–36.2	83	2.561	24.7	3.30	15.0–34.6
BCI_3_ = W/(SL*UG^b3^)	69	1.888	38.2	4.14	29.1–49.3	79	1.883	37.8	3.27	31.0–49.2
**Antillean manatee (*T. m. manatus***)*—Riverine										
BCI_1_ = UG/SL^b1^	47	0.934	0.69	0.08	0.41–0.94	43	0.854	0.66	0.09	0.28–0.78
BCI_2_ = W/SL^b2^	42	2.904	20.8	3.06	15.0–26.7	39	2.839	20.6	3.42	12.7–34.0
BCI_3_ = W/(SL*UG^b3^)	40	2.001	38.2	8.66	20.9–70.1	37	1.996	39.5	9.40	29.6–73.1
**Florida manatee **(***T. m. latirostris***)**										
BCI_1_ = UG/SL^b1^	63	1.045	0.72	0.04	0.64–0.84	83	0.844	0.86	0.04	0.79–0.97
BCI_2_ = W/SL^b2^	63	2.915	23.2	2.4	18.9–29.6	83	2.578	29.8	2.4	24.6–37.3
BCI_3_ = W/(SL*UG^b3^)	63	1.815	42.9	2.7	36.6–57.0	83	1.835	40.6	1.8	36.1–44.7

* This study, **Harshaw et al*.* 2016.

## Discussion

The development of efficient tools to appraise the body condition of manatees is necessary to advise monitoring and management actions to protect manatee populations. In this study, we developed and compared three morphometric body condition indices for Antillean manatees. Our results demonstrate that our three BCIs are suitable for the subspecies, with BCI_1_ (umbilical girth/body length) being easier to fit as it does not require measuring body weight, which can be challenging to collect in the field. It was also the most sensitive to Habitat influence, and has the potential to be sensitive to the detection of obese or malnourished animals. Comparisons of BCI_1_ among locations indicated differences in body condition between manatees living in freshwater ecosystems to those inhabiting coastal and marine areas, reinforcing that the subspecies *Trichechus manatus manatus* is likely comprised of, at least, two different ecotypes.

We compiled data collected by many researchers from thousands of hours of effort devoted to rescuing and studying Antillean manatees along a large distributional range and over decades. Our resulting database is to date the most comprehensive database of biometric information for the subspecies. Since most live manatee captures were conducted to equip the animals with remote monitoring telemetry tags, adult individuals were targeted and the proportion of calves is relatively small in our database (18%). However, the three BCIs met the important assumptions of lacking a correlation with standard length indicating that all of them may be suitable for all life stages.

For our analyses, we discarded manatees considered abnormal according to the in situ visual body condition assessment performed by the expert in charge (*i.e.,* C1, C2, C4, and C5 categories), and excluded females in the third trimester of pregnancy and one female from Brazil that was atypically large. This tool can aid in possibly determining pregnancy during late-stage development as the subject’s values may be an outlier to the expected range, but as already noted, the BCI for thin, obese, or pregnant manatees was generally within the normal range, so caution is warranted for using this calculation alone without other supporting evidence.

Florida manatees are generally larger than Antillean manatees as has been already reported^27,30,37^, with Florida manatees reaching a length of 376 cm and weighing up to 1620 kg^38^, and Antillean manatees reaching a maximum of 330 cm in length and 550 kg of weight (this study). Manatees with greater surface-area-to-volume ratio—*i.e.,* smaller in size and volume—would be more susceptible to develop cold stress syndrome, suggesting that cold winter water temperatures in Florida may have been an important selection factor for the larger body shape and size of the Florida subspecies^39^. Therefore, in response to conditions found in Florida, natural selection has not only increased the body size of Florida manatees, but also altered its body shape in relation to Antillean manatees^40^ with an overall proportionately larger girth. This supports Bergmann’s rule, which states that body sizes of individuals of a species inhabiting cold regions tend to be larger than those living in warmer regions^41^. Here, we demonstrate the need for normal BCI ranges for Antillean manatees, and suggest that similarly unique ranges may be found for the Amazonian manatee *Trichechus inunguis* and African manatee *T. senegalensis*. Since round trip movements by Florida manatees between the United States and Cuba have been already documented^42–44^, it would be interesting to explore body condition indices of Antillean manatees captured in the north of Cuba.

Our results show that the three BCIs fit well, and provide a solid base for estimating body condition for Antillean manatees. However, we gathered a significantly larger sample for BCI_1_ (UG/SL) because it does not depend on obtaining animal weight, which is logistically difficult to collect in the field. Although body mass is often needed for some energy related studies^45^, for some species this parameter may not always be easy to obtain during specific life-history stages or under particular conditions^46^. In many cases, manatee researchers in the field do not have the required equipment or logistic capacity to weigh manatees; and the volume of the individual is estimated by the circumference at the umbilical region. The best fit to weight was BCI_3_, which was also insensitive to Habitat, Sex, and body condition (obesity) and can be used to estimate weight when SL and UG are available. These two measurements are easily obtained during manatee handling. Remote body condition estimates can even be obtained through aerial photogrammetry, since SL can be measured directly, and UG can be calculated from the animal’s width^22,47^.

The weight of Antillean manatees can be estimated by using the following allometric equation:

9 $$W= {BCI}_{3}\times SL\times {UG}^{b3}$$

where W is the weight in kilograms, SL the straight-line total length in meters, and UG is the circumference at the level of the umbilical scar in meters, or:

10 $$W=37.67\times SL\times {UG}^{1.893}$$

for an average manatee across all Habitat and both Sexes. Values from Table 3 can be substituted for individuals of known Habitat and Sex, but BCI_3_ was relatively insensitive to these factors, and the only significant deviation from the mean was found for thin individuals.

Female Antillean manatees in this data set indicated a slightly smaller (non-significant) BCI_1_ than males, which was opposite to that found in Florida manatees^22^ but similar to dugongs^48^. The average female weight was slightly more than the average male weight (252 vs. 246 kg), and although these data do not constitute a random sample of all weight classes, they do follow the commonly observed pattern that *Trichechus manatus* sexual dimorphism is biased towards a larger body size in females. In aquatic mammals, a large body size may be an advantage in regard to defending against predators^49^, to store more oxygen and hence improve dive or apnea capacity^50^, to limit heat loss in the aquatic environment since large-bodied species have smaller surface-to-volume ratio^41^, and in the case of sirenians, a large body could also be attributed to their herbivorous diet^51^. According to a recent review^52^, sexual dimorphism appears to be a side effect of an adaptive increase in the body size of the species, and very often is linked to a polygyny reproduction system. In mammals, males are typically larger than females which is commonly associated with intra-sexual male competition^52^, Factors underlying the evolution of reversed sexual size dimorphism are poorly understood^53^, but it is possible that a larger female size in manatees has been selected to better resist male harassment in their polygyny reproduction behavior. Also, adult female manatees generally show less traveling^54^ and lower movement rates than males^55^, likely allowing them to accumulate more lipid storage necessary to support gestation and lactation periods.

**Figure 6:**
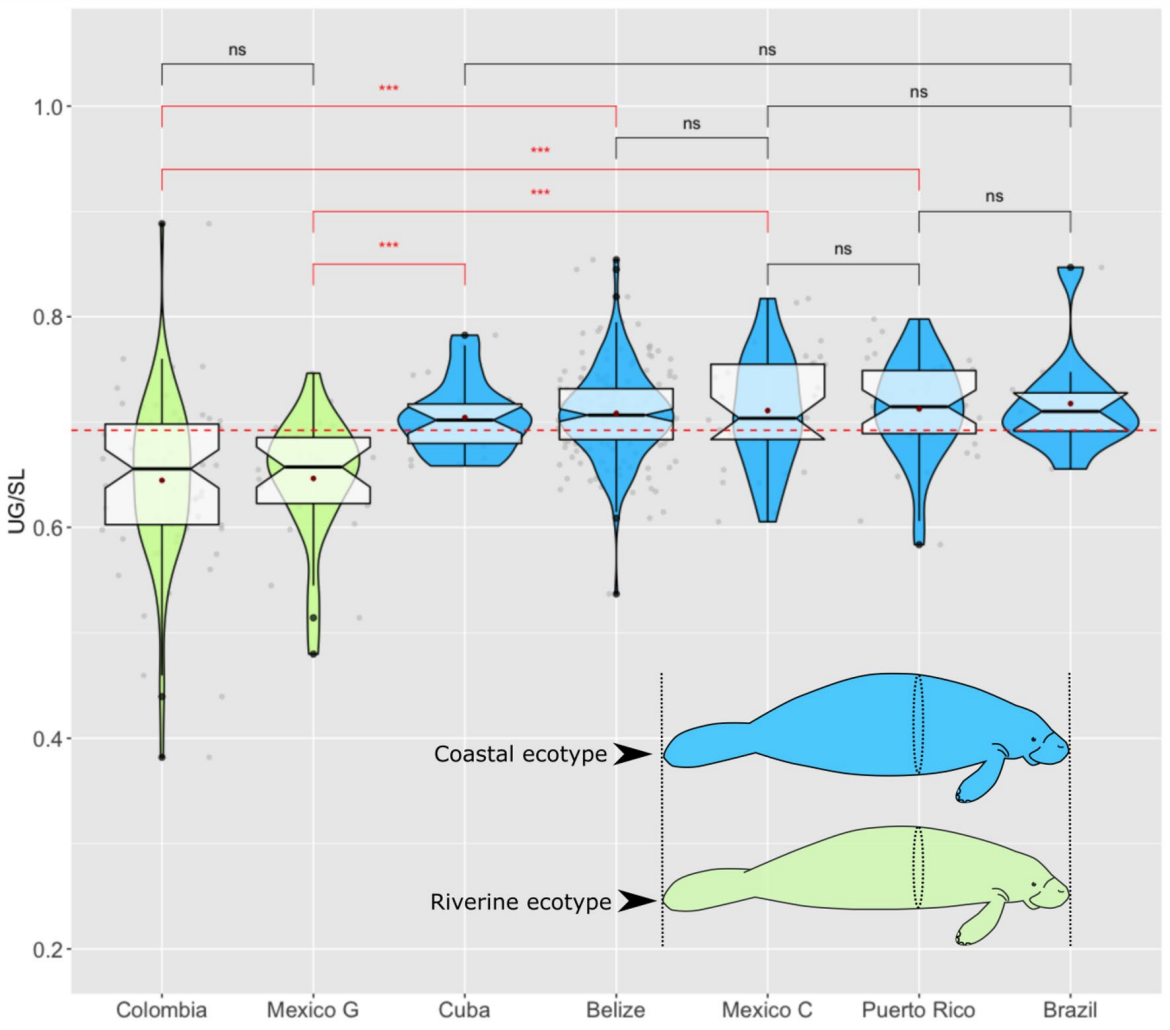
Violin plots showing significant differences in body condition (BCI_1_ = UG/SL) among Antillean manatees captured in two different environments: floodplain riverine systems (green) and coastal areas (blue). The red dotted line indicates the global mean of the BCI value (*n* = 362). Breaks represent examples of some *post-hoc* pairwise comparisons (Significance codes: *** = *p* < 0.0001, ns = no significant).

*Post-hoc* tests applied to BCI_1_ identified strong similarities between manatees from Colombia and Mexico G, but these two localities were statistically different from the other five locations (Table 6, Fig. 6). Manatees captured in Colombia and Mexico G inhabit predominantly freshwater environments consisting of complex systems of rivers, floodplains, and lagoons with a clear seasonal flood-pulse^56–58^. Individual manatees captured in the other localities (Puerto Rico, Cuba, Mexico C, Belize, and Brazil) occupy mostly coastal marine and estuarine environments, although they commonly make repeated trips to freshwater rivers to drink^59–61^.

**Table 6:** Pairwise comparisons among localities for BCI_1_ using Wilcoxon rank sum test with Bonferroni continuity correction.

		Riverine		Coastal marine			
		Colombia	Mexico G	Belize	Brazil	Cuba	Mexico C
Riverine	Mexico G	1					
Coastal	Belize	< 0.0001	< 0.0001				
	Brazil	0.0359	0.0046	1			
	Cuba	0.0023	0.0012	1	1		
	Mexico C	0.0003	0.0018	1	1	1	
	Puerto Rico	< 0.0001	< 0.0001	1	1	1	1

Habitat selection by Antillean manatees is strongly related to the availability of food, freshwater, and shelter, factors that vary differently depending on the environment they inhabit. Freshwater availability partially depends on the rainfall variation and appears to be the main factor influencing the movements of Antillean manatees living in coastal marine environments^60,62^. In contrast, manatees living in flood-pulse river ecosystems travel in response to the water level fluctuation^63–65^. During the low-water seasons, feeding resources for riverine manatees drop dramatically, and may force manatees to undergo periods of relative fasting in lagoons that become isolated during this period^66^. Thus, manatees living in areas such as the Usumacinta (Mexico G) and Magdalena (Colombia) river basins may have intermittent restrictions to food access during the year which can eventually negatively affect their overall body condition. Seasonal environmental stimuli may elicit endocrine responses of the organism: the increase of the ghrelin hormone during fasting or reduced nutrient intake stimulates the release of growth hormone, and inhibits lipids storage and gain in body weight^67^. For example, Florida manatees tend to show a reduced growth hormone, greater insulin-like growth factor hormone and greater fat thickness during short photoperiods (winter season)^68^.

Another important factor influencing manatee body condition is the nutritional value of their diet. Manatees in coastal marine environments consume primarily seagrasses, algae, and in smaller proportions, mangrove and other vascular plants^69–73^ with no evidence of seasonality in dietary composition^69^. In rivers, manatee diet includes a larger variety of plant species^74^, with a higher proportion of terrestrial plant consumption, and a clear seasonality in diet composition^75^. Previous research indicates that seasonal limitations in plant growth leads to altered nutritional composition of aquatic plants^76^, a condition that can occur in riverine systems during the dry season and may affect the overall fitness of individuals. West Indian manatees have a slow digestive passage rate^77–79^, efficient decomposition of fibrous material through microbial degradations^79^, and high digestive efficiency^80^. According to a recent study, low-fiber manatee diets may be more digestible because they have less lignin content^81^. Thus, since marine angiosperms have low lignin values when compared with terrestrial angiosperms^76^, it can be expected a greater digestibility in manatees living in coastal marine environments when compared to manatees that feed on terrestrial or freshwater aquatic plants. More detailed stable isotopic studies on tissues could shed more light on the specific use of varying habitats among manatee populations^71,75,82,83^. Details on the variation in the bioenergetics of the subspecies according to their diets are needed to elucidate the physiological implications of their digestion.

Our results open an interesting discussion about the phenotypic plasticity of the subspecies and suggest that a single genotype may have originated at least two alternative forms^84^ with differing behavior (*e.g*., habitat use) and morphology (*e.g.*, robustness) in response to differences in environmental conditions. More information on genotypic variation, habitat use and feeding habitats would be informative to support this hypothesis. The two ecotypes—riverine and coastal Antillean manatees—may face different fitness tradeoffs relative to environmental and resource limitations, influencing ultimately the calculated BCIs.

The overall health assessment during an examination of a manatee consists of several factors and tools in order to completely determine the condition of the population by assessing a few individuals^85^. The BCI is one of the tools in the arsenal, but does not provide a complete picture of the health status of the animal nor concrete evidence of its condition, thus other ancillary information should be collected. Blood work is critical, and customary tissues should be collected and properly archived in the event that additional studies may be required. Researchers typically assume that body condition index is a proxy of lipid content, which in turn is supposed to be positively and directly related to fitness or some component of fitness^86^. However, this is not always the case, and caution should be taken to not over interpret the usefulness of BCIs for manatees. Nevertheless, to carry this further, using BCI can inform researchers of the nutritional status of the manatees handled in the future.

Biometric body condition indices are often considered composite metrics of nutritional physiology, physical status, and health^86^, allowing the integration of ecologically relevant aspects^87^. In the long term, body condition of wild manatees can be a valuable parameter to evaluate the impact of several environmental stressors^88^, and to advise management strategies^89^. For example, BCIs can be used to assess the impact of stress level as a response to changes in manatees’ habitat that disrupt access to food and/or freshwater, and exposure to contamination and other persistent human-related disturbances. This will ultimately serve to inform development of sound management plans and guide regional based efforts to help conserve the subspecies.

## Supplementary Information

Supplementary Information.
