# Supplementary material for: Analysis of body condition indices reveals different ecotypes of the Antillean manatee
Source: Sci Rep. 2021 Sep 30;11:19451. doi: 10.1038/s41598-021-98890-0 (PMC8484672; doi:10.1038/s41598-021-98890-0)
Supplement: Supplementary file 1 — Supplementary Information. [file 41598_2021_98890_MOESM1_ESM.docx]

**Supplementary material 1.** Illustration of lateral, dorsal and ventral views of the 5 veterinary body conditions based on clinical observation as in Supplementary material 1.


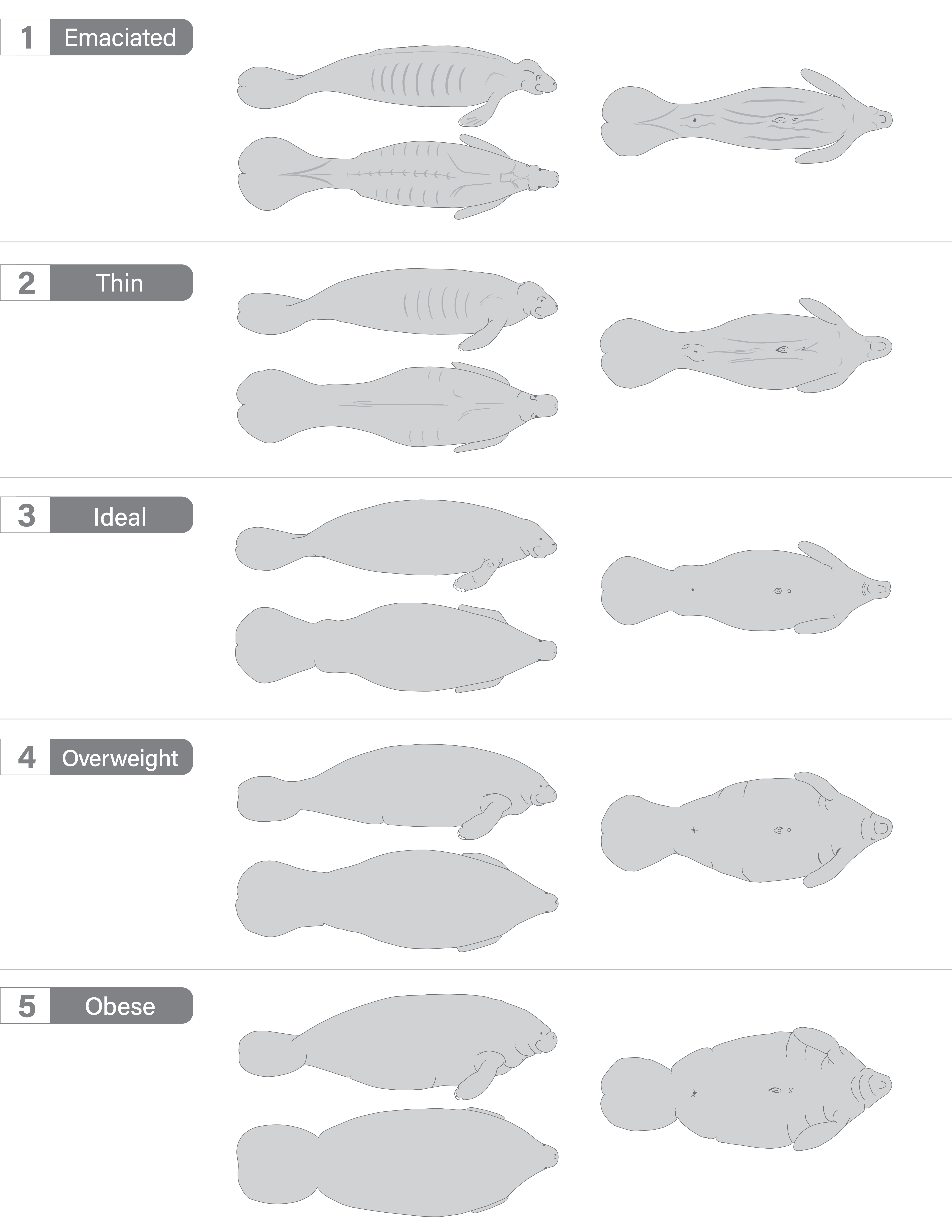


Supplementary material 2. Evaluation of body condition categories on Antillean manatees based on clinical observations.

|  | **Emaciated (C1)** | **Thin (C2)** | **Ideal (C3)** | **Overweight (C4)** | **Obese (C5)** |
| --- | --- | --- | --- | --- | --- |
| **General Description** | Pronounced loss of body mass and wasting of epaxial muscles becomes evident with marked and evident bone structures. | A slight loss of body mass is observed. Bone structures begin to be evident. | The body acquires a fusiform and robust shape with a good body mass typical of a Caribbean manatee. No marked bone structures are evident. | The body's robust shape becomes heavier and rounder. | Body is extremely oval/round, with massive body mass and an absence of body part boundaries. |
| **Head** | Loss of muscle mass around the skull, with a marked bony ridge dorsal to each eye. The temporal bones are visible. | Thin muscle mass around the skull, with a visible bony ridge dorsal to each eye. | Palpable muscle mass around the skull, with a light bony ridge dorsal to each eye. | Moderately convex head. Bony ridge dorsal to each eye begin to disappear. | Heavy convex and robust head, with the shape of the skull completely erased and only the eyes, nostrils and snout are visible. |
| **Neck** | Marked thinner neck with highly deliniation between head, neck, and trunk. A distinct "peanut-head" is apparent. | Thinner neck with observed deliniation between head, neck and trunk. A "peanut-head" begins to be observed. | Slightly rounded with a slight differentiation between neck and head | Robust neck with a moderate marking between neck and head. | Extremely robust and rounded neck with rolls of fat. No delimitation can be observed between head, neck, and trunk of the manatee. |
| **Trunk** | The trunk is ventrally flattened, with excessive loss of muscle mass. Ribs, scapula and vertebral column become noticeable evident. | The trunk becomes flatter ventrally with lighter muscle mass. Ribs and scapula become insinuated. | The trunk is slightly oval and fusiform, with good muscle mass. | The trunk's shape becomes rounder. | The trunk is excessively round, taking a typical "potato" shape. |
| **Peduncle** | The peduncle is thin with a noticeable loss of muscle mass. A "V" shape is observed as the caudal vertebrae begin to show. | The peduncle is slightly thinner and loses rolls of fat connecting to the tail. | The peduncle connects with a few rolls of fat to the tail. | The peduncle becomes thicker as rolls of fat become fuller connecting to the tail. | The typical peduncle shape is lost as it becomes so thick that there is no smooth transition to the tail. |

|  | **Emaciated (C1)** | **Thin (C2)** | **Ideal (C3)** | **Overweight (C4)** | **Obese (C5)** |
| --- | --- | --- | --- | --- | --- |
| **Forearm** | Demarcation of scapulae, flippers and flipper’s hand are evident. No fat folds observed in axilla. | Scapula and bones of the flippers are moderately visible with the movements of the individual. Axilla fat folds become less visible. | Scapulae are only visible when manipulating the flippers. Axilla has observable folds of fat. The fingers on the flippers are barely visible. | A roll of fat begins to form around the shoulder. Axilla fat folds are more evident. The flippers become noticeably thicker and start to loose mobility. | Scapula not observed. Large folds of fat wrap around the shoulder, axilla and forearm, restricting the natural mobility of the flipper. |
| **Dorsal view** | A visible thin body with highly marked head, trunk and peduncle. Skull, vertebral column, ribs, and scapula are markedly visible and palpable. Caudal vertebrae form a "V" shape. The snout looks longer. | The oval shape becomes thinner, as the head, trunk and peduncle obvious. Vertebral column, ribs and scapula are slightly visible. | The body has an oval shape with a slight differentiation between head, trunk and peduncle. Vertebral column and ribs are not visible. | The body's oval shape become robust, and differentiation between head, neck and peduncle begin to disappear. | Extremely robust barrel-shaped body with thickened neck and peduncle. The only differentiation is that the head and snout are small compared to the enlarged body. |
| **Lateral view** | Visibly thin with flattened dorsum and ventrum, with highly marked head, neck depression, trunk and peduncle. Ribs and a "peanut-head" are clearly visible. | The oval shape becomes flatter dorsally and ventrally, and the head, neck depression dip, trunk and peduncle become markedly visible. Ribs and a "peanut-head" are slightly visible. | The body has an oval shape with a slight differentiation between head, trunk and peduncle. Ribs are not visible. | A more robust body with little differentiation between head, trunk and peduncle. Dorsal neck depression becomes less apparent and a slight dewlap begins to develop. | Extremely robust and rounder body, with no neck depression or peduncle differentiation, except for a small head. Dewlap is remarkable. |
| **Ventral view** | There is a severe concavity of the trunk. Multiple abdominal folds form around the genitourinary apertures and anus. Caudal vertebrae form a "V" shape. The mandibular ramus is highly visible. | The trunk takes on a flat shape with a slight concavity. Ventral folds become evident around the genitourinary apertures and anus. | Slightly convex trunk and peduncle. Light dewlap in ventral neck region. | Trunk becomes highly convex with an enlarged neck dewlap. Rolls begin forming around the axilla causing the flippers to become rigid. | Trunk is extremely convex as the neck dewlap merges with the chest. Enlarged rolls around the axilla limit the natural movement of the flippers. |
